# Isolated prostatic utricle

**DOI:** 10.4103/0971-9261.59610

**Published:** 2009

**Authors:** Mukunda Ramachandra, Pradnya S. Bendre, Rajeev G. Redkar, Devendra V. Taide

**Affiliations:** Department of Paediatric Surgery, B.J.Wadia Hospital for Children, Parel, Mumbai, India

**Keywords:** Mullerian duct remnants, posterior sagittal approach, prostatic utricle

## Abstract

Complete excision of a prostatic utricle through posterior sagittal rectum retracting approach is reported in an infant.

## INTRODUCTION

Persistent mullerian duct tissue in male individuals may result in enlarged prostatic utricle. In disordered sexual differentiation, mullerian duct remnants are common. Isolated utricles in male individuals are not common in clinical practice. Surgical management remains challenging because of the close proximity of these lesions to the ejaculatory ducts, pelvic nerves, rectum, vas deferens, and ureters.[1]

## CASE REPORT

**Figure 1:**
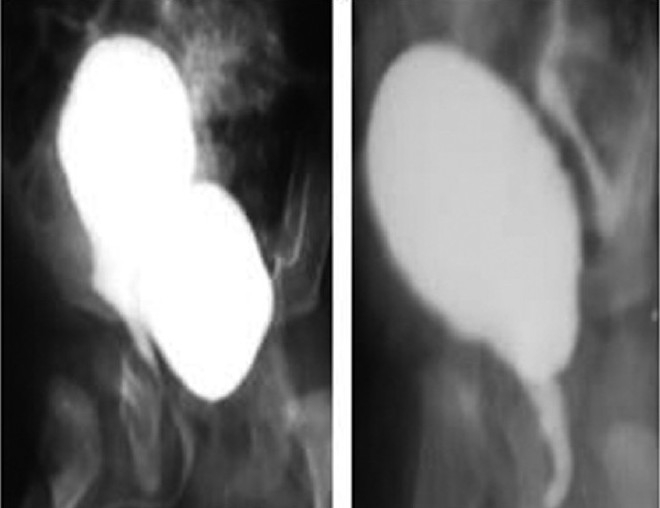
Pre-operative and post-operative MCU

A 6-month-old boy presented with retention and poor stream of urine since birth. There was no history suggestive of repeated urinary tract infections or failure to thrive. External genitalia was normal. Antenatal sonography was normal. Abdominal examination revealed palpable bladder and on rectal examination bladder was thickened. On ultrasound of the abdomen, there was no hydronephrosis and hydroureter. Micturating cystourethrography (MCU) showed good capacity bladder with crenations, grade II prostatic utricle, thin stream of urine [Figure 1], and significant postvoid residue. By posterior sagittal rectum retracting approach (PSRR), the prostatic utricle was completely excised.

## OPERATIVE TECHNIQUE

Cystoscopy was done and a ureteric catheter placed in the utricle. The patient was catheterized besides the ureteric catheter and placed in the prone jack knife position. A large bore red rubber catheter was placed in the rectum. A midline incision was taken from the coccyx to about 1.5 cm posterior to anus. It was deepened in the midline, dividing the parasagittal fibers and levator ani without cutting external sphincter, with the help of muscle stimulator until the rectum is reached. The investing layer of rectum was divided in the midline and rectum retracted onto the patient's right side with the help of Deaver's retractor. The utricle was identified with the help of ureteric catheter, which is anterior to urethra and posterior to rectum in the center of the wound. Stay sutures were taken on the utricle and dissected free from the urethra, seminal vesicles, and vas, which are easily identifiable with this approach, thus avoiding injury to these important structures. Utricle was dissected all around and to its junction with the urethra and excised in flush with the urethra without leaving any diverticulum. The urethral opening is closed with 4.0 vicryl. The rectum is allowed to fall back into position and the wound was closed in layers with a glove drain. Bladder catheter was kept for 5 days.

## FOLLOW UP

The child had uneventful postoperative recovery. MCU done after removal of bladder catheter showed complete excision of the utricle. The child did not have any voiding dysfunction (12 months follow-up).

## DISCUSSION

Prostatic utricle is a median epithelium lined sinus 4-6 mm long, opening between the two ejaculatory ducts on verumontanum. It represents the caudal end of fused mullerian duct corresponding to vaginal and cervical portion of duct.[2] The mullerian duct is present in all human embryos at the early stages of development. In male subject, secretion of mullerian inhibiting substance causes regression of mullerian system. Utricular anomalies result from the incomplete regression of mullerian ducts or incomplete androgen-mediated closure of the urogenital sinus caused by an error in the production of or sensitivity to local testosterone or mullerian inhibiting substance.[3] This explains prostatic utricle among various stages of inter-sex, hypospadias, and cryptorchidism.

Many utricles are asymptomatic and, therefore, escape cryptorchidism detection. Enlarged utricle may present clinically as lower urinary tract irritative symptoms, postvoid dribbling, urethral discharge, repeated UTI, stone formation in the pouch or pseudo incontinence due to secondary trapping of urine in the pouch, and retention of urine. An enlarged utricle can be discovered by inadvertent catheterization of utricular orifice during hypospadias repair. Schuhrke and Kaplan[4] noted a 3% incidence of malignancy in prostatic utricle.

Enlarged utricle can be readily appreciated by retrograde urethrography or MCU. Ikoma *et al*.[5] proposed a grading system as seen on MCU: grade 0 – opening located on the urethra but the utricle does not extend over the verumontanum; grade 1 – larger than grade 0, but it does not reach bladder neck; grade II – more enlarged and its dome extends over the bladder neck. The prostatic utricle opens into the central area of the verumontanum in the prostatic urethra in grade 0, I, and II. In grade III, the opening is situated in the bulbous urethra just distal to the external sphincter.

Surgical excision is the definitive treatment of symptomatic enlarged prostatic utricle. Disorders in this area are considered too high to be approached through the perineum or too low to be approached through the abdomen. Many approaches have been described. Schuhrke and Kaplan[4] reported endoscopic transurethral cyst catheterization and aspiration, cyst orifice dilatation, incision, or unroofing. Ahmed and Palmer reported successful transperineal cyst aspiration and sclerotherapy by tetracycline under transrectal ultrasound guidance.[6] Willetts *et al*.[7] reported successful laproscopic excision of an utricle in a child. Unfortunately endoscopic and laproscopic approach have technical limitations.

Open excision is the better definitive treatment in the pediatric age group. Perineal, suprapubic, extravesical, transperitoneal, parasacral, transvesical transtrigonal, retropubic, transanorectal posterior, or anterior sagittal approaches have been described. However, all require extensive dissection, sometimes having two stages and often result in poor exposure. The most severe complications include inadvertent injury to pelvic nerves causing incontinence. The efforts sometimes ended in incomplete excision, and complicated dissection often needed the excision of one or both seminal vesicles, vas, and portions of the prostate. PSRR approach have good exposure of posterior urethra, exact visualization of all important structures, and complete excision of prostatic utricle.^[8]^

## References

[1] Desautel MG, Stock J, Hanna MK (1999). Mullerian duct remnants: Surgical management and fertility issues. J Urol.

[2] Oh CS, Chung IH, Won HS, Kim JH, Nam KI (2009). Morphologic variations of the prostatic utricle. Clin Anat.

[3] Schuhrke TD, Kaplan GW (1978). Prostatic utricle cysts (Mullerian duct cysts). J Urol.

[4] Ikoma F, Shima H, Yabumoto H (1985). Classification of enlarged prostatic utricle in patients with hypospadias. Br J Urol.

[5] Husmann DA, Allen TD (1977). Endoscopic management of infected enlarged utricle and remnants of rectourethral fistula tracts of high imperforate anus. J Urol.

[6] Willetts IE, Roberts JP, MacKinnon AE (2003). Laparoscopic resection of a prostatic utricle in a child. Pediatr Surg Int.

[7] Meisheri IV, Motiwale SS, Sawant VV (2000). Surgical management of enlarged prostatic utricle. Pediatr Surg Int.

